# THE INFLUENCE OF EDUCATIONAL DANCE ON THE MOTOR DEVELOPMENT OF CHILDREN

**DOI:** 10.1590/1984-0462/;2018;36;3;00004

**Published:** 2018

**Authors:** Isabelle de Vasconcellos Corrêa dos Anjos, Alexandre Archanjo Ferraro

**Affiliations:** a Universidade de São Paulo, São Paulo, SP, Brasil.

**Keywords:** Child development, Child, School health, Dancing, Motor activity, Psychomotor performance, Desenvolvimento infantil, Criança, Saúde escolar, Dança, Atividade motora, Desempenho psicomotor

## Abstract

**Objective:**

The purpose of this study was to compare the motor development of children who practiced educational dance with the motor development of children who did not practice it and to verify the results obtained after six to eight months after the end of the intervention.

**Methods:**

The study was carried out with 85 children enrolled in the first year of elementary school in two schools located in the south of São Paulo city (São Paulo, Brazil). Children were randomized by lot in two groups (intervention and control). Children with intellectual and/or physical disabilities and the premature ones were excluded from the analysis. The two groups had their motor development evaluated in three moments: before the intervention, after the intervention and six to eight months after the end of the intervention. The intervention group participated in an educational dance class program for seven months. Control and intervention groups were compared by chi-square and t-test.

**Results:**

Children who participated in the educational dance program, compared to children who did not, achieved significant gains in their general motor development and on the following bases: balance, fine motor and overall praxis.

**Conclusions:**

Educational dance helped the children’s motor development, and the results were partially maintained months after the end of the intervention.

## RESUMO

**Objetivo:**

A proposta deste estudo foi comparar o desenvolvimento motor de crianças que praticaram dança educativa com o desenvolvimento motor de crianças que não a praticaram e verificar a permanência dos resultados obtidos, após seis a oito meses do término da intervenção.

**Métodos:**

O estudo foi realizado com 85 crianças matriculadas no 1º ano do ensino fundamental de duas escolas da zona sul de São Paulo (SP), randomizadas por sorteio em dois grupos (intervenção e controle). Foram excluídas da análise as crianças com deficiência intelectual e/ou física e as prematuras. Os dois grupos tiveram seu desenvolvimento motor avaliado em três momentos: antes da intervenção, após a intervenção e depois de seis a oito meses do término da intervenção. O grupo intervenção participou de um programa de aulas de dança educativa por sete meses. Os grupos controle e intervenção foram comparados por teste qui-quadrado e teste *t*.

**Resultados:**

Os resultados indicaram que as crianças que participaram do programa de dança educativa obtiveram ganhos significativos em seu desenvolvimento motor geral e nas bases: equilíbrio, praxia fina e praxia global, em comparação às crianças que não participaram desse programa.

**Conclusões:**

A dança educativa auxiliou na evolução do desenvolvimento motor de crianças, e seus resultados mantiveram-se parcialmente meses após o término da intervenção.

## INTRODUCTION

A study^1^ showed that in the population so-called “normal”, 35% of the children enrolled in the 1^st^ year of elementary school show developmental delay, and, among the population groups with social vulnerability, such estimation reaches 46%. When motor development is considered, contemporary challenges are observed. Currently, most children do not play on the streets; their favorite games do not require movements, and are mostly on cell phones, tablets, videogames etc.^2^ Santos and Vieira^3^ showed that motor development delay and coordination disorder are highly prevalent, which is a matter of concern, since these findings are associated with negative impacts on social, emotional, affective and school relations of the children. The transition from child education to elementary school increases the challenges and requires more adaptation, motor control and skills. Children spend more time sitting down, need to pay attention for longer periods of time, and their cognitive development becomes the focus.^4^

School is a privileged environment to observe child development and to intervene in it. Attending daycare facilities/school in the first five years of life generates benefits that are not only educational, but also related to health. Campbell et al.^5^ reported that children attending the educational structure at that age are exposed to more stimuli and interventions for their development, and present better physical health after the age of 30.

The literature reports intervention studies that aimed at improving motor development in the age group attending elementary school. Babin et al.^6^ found improved motor aptitude in children enrolled in the 1st grade of elementary school in Croatia, after the implementation of a physical and health education program in the Physical Education classes. Krneta et al.^7^ pointed out to significant improvement in the development of motor skills of children in pre-school, with kinesiological activities (stretching, exercises requiring muscle strength etc.). Sawada et al.^8^ observed higher efficacy in learning dance movements when the instructions were metaphorical, instead of isolated verbal instructions or movements; however, they did not aim at measuring the motor development resulting from the dance practice.

Facing the exposed, more studies are required to test if dancing in school presents itself as a positive instrument for motor development, since unlike other areas and techniques, dancing prioritizes experimentation and promotes creativity, working with emotions, interacting with other people, music and the body itself, therefore providing self-knowledge and the ability to overcome limitations.

Therefore, the objectives of this study were to compare the motor development of children aged from five to six years who practiced educational dance with the motor development of children at the same age who did not practice it, and verify the permanence of the results obtained six to eight months after the conclusion of the intervention.

## METHOD

This was a randomized intervention study, in which one group attended two one-hour dance classes a week, for seven months. The other group was control and did not attend any dance classes. Both groups maintained identical numbers of Physical Education and Art classes, according to the official program of the State of São Paulo. Randomization was conducted per group (n=4), by a raffle carried out by a person who was not part of the study.

The population analyzed included students enrolled in the 1st year of elementary school from two institutions of the Center-West Educational State Network of São Paulo, located in the same district. Both schools are in the Itaim Bibi neighborhood, and include only the segment of the early years of elementary school, that is, form the 1^st^ to the 5^th^ grades. The students were mostly from the Paraisópolis neighborhood, approximately seven kilometers away from the schools, but, to have access to the vacancies, their parents/tutors worked close to them. School vans transported the children and were provided by the City Hall.

Randomization was conducted by class, and not by individual, so that there would be no risk of contamination of the intervention over the subjects in the control group. Besides, the children might feel excluded from the group to which they already belonged. All students enrolled in the four 1^st^ grade classes (two classes in each school) were eligible for participation; however, only those whose parents and/or tutors signed the Informed Consent form participated in the activities.

The exclusion criteria were applied only at the time of the analysis, according to the questionnaire filled out by the parents/tutors, and were listed as follows: presenting physical or intellectual disabilities (remarkable reduction in intellectual and/or physical function, significantly lower than average); practicing extracurricular physical activities (frequent activities carried out in alternate shifts in relation to regular school hours); and children who were born premature.^9^

The intervention was based on the methodology created by Rudolf Laban,^10^ called educational dance, whose starting point are the natural movements of each person. Then, with creative and ludic proposals, it stimulates the participants to discover and experiment new movements, new ways of performing the movements they already know, besides knowing their own limitations and improving their interpersonal relationship. Laban^10^ defined that all movements have combinations of nuances including four factors: weight, time, space and fluency. These combinations result in basic actions (slide, push, float, whip etc.). The study also contemplates the levels of movement (high, medium, low), kinesphere (room occupied by the body in space), among other concepts. For the experimentation of these concepts, ludic games and representations are developed, starting with the movements that are already known, enabling the discovery of new movements and possibilities. The lessons were ministered by a professional specialized in the Laban methodology, who planned the activities with progressive levels of difficulty, synchronized with the specific developmental moment of each class. The focus were the movement factors, but also other items related with the methodology: levels of movement, kinesphere, and some basic actions. The activities were carried out for seven months, in one-hour lessons, twice a week, in the classroom, court or patio, isolated from other classes. These structures were granted by the schools, depending on the logistic possibilities of the day of the lesson.

The outcome measurements (motor development - MD) were taken based on the Motor Development Scale created by Francisco Rosa Neto^11^ and validated by Rosa Neto et al.^12^ ^,^ ^13^ The scale measures whether the MD of the children coincides with, is higher or lower than the MD of their chronological age, in months. This assessment has tests for each psychomotor base: tonus, balance, lateralization, body notion, space-time structure, global praxis and fine praxis. Each test contains tasks defined by age, with levels of difficulty and gradual increase. The result of each test generates a score, which is characterized as: superior, normal-high, normal-medium, normal-low, inferior, very inferior. The mean score of the tests follows the same categorization. The evaluations were carried out in three moments: pre-intervention; immediately after the conclusion of the intervention; six to eight months after the intervention. The evaluations were conducted by an external evaluator who did not know if the children were in the control or in the intervention group. 

The data collected in the beginning of the intervention were: parental schooling and age, number of siblings, time of gestation, weight and length at birth, time of breastfeeding, of no longer wearing diapers, pre-existing or current diseases, sitting-down, crawling and walking age, current weight and height,^14^ as described in Table 1.

**Table 1:** Characterization of the studied sample.

	n	Mean±standard deviation
Mother’s age (years)	83	30.9±7.0
Father’s age (years)	83	35.3±6.7
Weight at birth (kg)	83	3.0±0.4
Body mass index (kg/m^2^)	83	14.3±0.4
Gestation (months)	83	9.0±0
Length at birth (cm)	83	47.0±2.7
Breastfeeding (months)	83	9.8±6.9
No longer wearing diapers (months)	83	24.0±9.0
Sitting down (months)	66	6.5±0.7
Crawling (months)	74	7.8±0.9
Walking (months)	74	11.8±1.7
Talking (months)	62	14.8±5.2
Number of siblings	83	1.8±1.0

**Figure 1:**
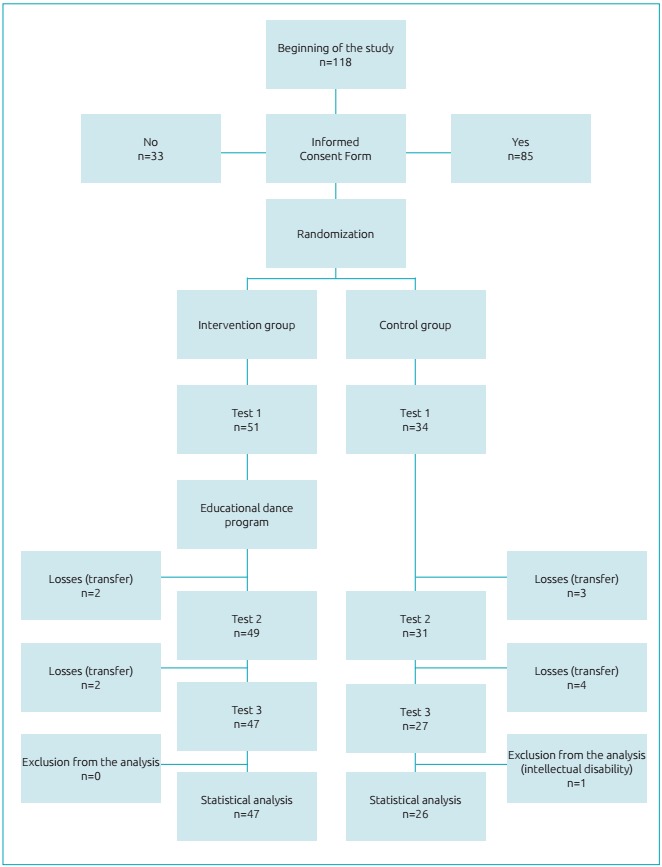
Direction of the study and number of children in each stage.

The calculation of the sampling size was based on the following assumptions: the expected difference between the intervention and the control group would be of 3 months at the age of motor development; the first species error would be 0.05 (1.96 in normal distribution); and the second species error would be 0.20 (0.84 in the normal curve), giving the sample an 80% test power. Eighteen students were estimated to be necessary in each group. Because the intervention was carried out during school time, the random allocation of the intervention/control groups was conducted in the classroom. That is, the raffle took place per class/group, and not per student, resulting in two intervention groups and two control groups. The intervention group was composed of 51 children, and the control group, 34. The difference in the number of children in each group was a result of the non-consent from parents/tutors in part of the group selected for control in one of the schools, with the religious justification that dancing was not allowed. There was no pairing, and to rule out the influence of confounding variables, both groups were compared in test 1 (before intervention), checking the balance of variables of interest. The sample is summarized in Figure 1.

Since the continuous variables had normal distribution, they were described as mean and standard-deviation. The categorical variables were expressed in number and percentage. The analytical study used the *t*-test to compare the means in the control and in the intervention groups, in the three moments, for each one of the psychomotor bases assessed. A Signal test was also conducted to compare the groups in the classification of the Motor Development Scale. Finally, the third evaluation took place in the period that ranged between 5.9 and 8.4 months after the conclusion of the intervention. In order to know if the effect of the intervention over the psychomotor outcomes was influenced in this period of time, we performed the linear regression with the psychomotor parameters, such as outcome and effect of the intervention, adjusted for the interval between the second and the third test, categorized in terciles.

The study protocol was approved by the Research Ethics Committee of the Medical School of Universidade de São Paulo, n. 069/13.

## RESULTS

To characterize the studied sample, a questionnaire was applied to parents/tutors in the period of the intervention, in both groups. Most analyzed children were born in natural labor and did not have any diseases reported by their parents. Most mothers had completed high school, but most fathers did not. The children’s diseases reported by some parents were: diabetes, rhinitis, asthma, allergies to insect bite and optical refraction conditions (Table 1). One child was reported as being in the autism spectrum disorder.

In the first test, both the intervention and the control groups were similar in relation to chronological age, body mass index (BMI) and sex, besides having presented similar results regarding motor development, indicating that randomization produced two comparable groups (Table 2). After the intervention, the group showed significant evolution in comparison to the control group, except in the bases: body scheme, spatial organization and temporal organization. After the third test, both groups had approximated their results; the intervention group maintained the gain obtained by the intervention, and the control group evolved as expected for their chronological age (Table 3).

**Table 2:** Comparison between the control and intervention groups in the first test, before intervention.

	Categories/unit	Control	Intervention	p-value
Male gender	n (%)	15.0 (44.1)	20.0 (39.2)	0.653**
Body mass index	kg/m^2^	17.2±0.5	17.4±0.4	0.841*
Chronological age	Months	78.8±4.2	77.7±3.9	0.234*
Motor development n (%)	Very inferior	2.0 (5.9)	5.0 (9.8)	0.313**
	Inferior	8.0 (23.5)	6.0 (11.8)	
	Normal-low	10.0 (29.4)	11.0 (21.6)	
	Normal-medium	13.0 (38.2)	23.0 (45.1)	
	Normal-high	1.0 (2.9)	6.0 (11.7)	
	Superior	-	-	

*p-value of the t-test; **p-value of the chi-square.

**Table 3:** Comparison of results of tests 1 (before intervention), 2 (immediately after intervention) and 3 (six to eight months after intervention), in the control and intervention groups in months, expressed in mean±standard deviation.

	Test 1		Test 2		Test 3	
	Control	Intervention	Control	Intervention	Control	Intervention
General motor age	67.70±10.3	68.60±10.3	76.30±10.5	85.10±11.4**	88.10±12.0	87.00±15.8
Positive age/negative age	-11.10±10.2	-9.20±10.6	-9.30±10.0	-0.10±11.0**	-5.80±12.6	-5.20±15.3
General motor quotient	86.00±13.0	88.30±13.6	88.90±11.9	100.00±12.8**	94.00±13.5	94.40±17.0
Fine motor quotient	90.30±21.2	91.00±19.9	87.90±23.7	102.70±25.1*	101.00±25.7	105.40±25.4
Global motor quotient	101.00±28.1	96.00±26.4	107.80±20.0	123.20±25.7***	112.74±19.4	107.30±27.9
Balance quotient	90.10±28.6	101.40±35.7	89.70±21.0	111.10±28.0*	83.80±19.8	91.20±28.1
Body scheme quotient	77.50±14.7	81.90±13.5	87.80±10.5	91.80±12.3	95.10±19.9	96.60±21.0
Spatial organization quotient	78.80±20.3	79.80±23.8	81.30±18.6	85.70±18.2	90.20±17.5	83.80±20.9
Temporal organization quotient	78.50±16.1	80.00±15.6	79.10±13.8	85.50±21.1	81.20±10.7	82.20±18.0

*T* test: *p=0.011; **p=0.001; ***p=0.006.

To understand the clinical impact of the changes found, Table 4 classifies the categories of the motor development of participants. It is observed that, in test 1, there were no differences between the groups; then, in test 2, there was statistically significant difference between those who took part in the intervention and the control group. The chi-square test does not show the level that mostly contributed with the difference found, but the distribution between categories shows that the intervention group had more children in better categories.

**Table 4:** Evolution of the classification of the Motor Development Scale, in tests 1 (before intervention), 2 (immediately after intervention), and 3 (six to eight months after intervention), in the control and intervention groups, by number of students (%).

	Very inferior	Inferior	Normal- low	Normal-medium	Normal-high	Superior	Total
Test 1							
Control	2 (5.9)	8 (23.5)	10 (29.5)	13 (38.2)	1 (2.9)	-	34 (100)
Intervention*	5 (9.8)	6 (16.5)	11 (24.7)	23 (42.4)	6 (8.2)	-	51 (100)
Test 2							
Control	1 (3.2)	5 (16.1)	12 (38.7)	11 (35.5)	2 (6.5)	-	31 (100)
Intervention**	-	3 (6.1)	7 (14.3)	27 (55.2)	9 (18.4)	3 (6.2)	49 (100)
Test 3							
Control	1 (3.9)	2 (7.7)	5 (19.2)	15 (57.7)	2 (7.7)	1 (3.8)	26 (100)
Intervention***	3 (6.4)	7 (14.8)	2 (4.3)	27 (57.5)	5 (10.6)	3 (6.4)	47 (100)

Control *versus* intervention; chi-square test: *p=0.313; **p=0.018; ***p=0.396.

After the adjustment by interval of time between the second and the third evaluation, there was no significant difference between the different psychomotor parameters, with the following means and 95% confidence intervals (95%CI): general motor age, -2.39 (-9.38-4.60); general motor quotient, -0.69 (-8.38-7.00); fine motor quotient, 2.74 (-9.65-15.14); global motor quotient, -6.73 (-19.19-5.73); balance quotient, 6.88 (-5.83-19.58); body scheme quotient, 0.62 (-9.49-10.74); spatial organization quotient, -7.34 (-17.22-2.47); and temporal organization quotient, -0.26 (-7.47-6.94). The absence of the intervention effect between the second and the third evaluation still existed, even after the model was adjusted for the interval of time between them.

## DISCUSSION

The results of the study showed statistically significant improvement in the motor development of students exposed to educational dance lessons, in comparison to the control group. In this study, both groups obtained positive results; however, the intervention group evolved faster. Right after the conclusion of the intervention, the children who took part in the program presented improvements in motor development and evolved in general motor age, being mostly included in the normal-medium, normal-high and superior classifications. After the conclusion of the program, they maintained this gain, however, with the advancement of chronological age, their results were in the categories normal-low, normal-medium and normal-high, whereas those in the control group evolved according to their chronological age, getting closer to the ones in the intervention group. We can state that the practice of educational dance should be more longitudinal, once motor development is permanently evolving. Its objective should not only be that of acquiring what would be expected overtime, but also improvement and discovery of new motor possibilities.

We consider this study to be of great importance. In some European countries, dancing is a mandatory part of the school curriculum. In Brazil, despite being part of the Regiment of the Municipal Secretariat of Education in São Paulo^15^ as a differential language since 1922, and being included in the National Curricular Parameters, published in 1997-98 by the Ministry of Sports and Education (MEC),^16^ in which it is mentioned and suggested as part of Arts Education,^17^ the dance still does not occupy the place it should have in schools. Recently, Law n. 13,278/2016 was sanctioned and includes dancing as a distinct field of knowledge, as well as theater, music and the visual arts.^18^ In spite of that, in the scientific literature, the existence or not of beneficial effects over health has not been properly proven.

In our field, the dance practice at school age presents itself in two forms: classic ballet, whose dancing technique does not include all students, for being extracurricular and usually paid for, and because of the negative cultural factor regarding the fact of boys dancing classic ballet; and choreographies performed especially for typical parties, such as June festivities, Mother/Father’s Day, spring and graduation, in which virtuosity and technical improvement are more valued than the ability of creation and reflection.

Studies state that cognitive development is related with motor development.^19^ ^,^ ^20^ This association has not been tested in this study, but if motor gain has also implied in cognitive gain, the benefits of the intervention may have been greater and more lasting than those we measured. The study design with randomized allocation in the intervention/control groups; the insertion of the intervention in the school curriculum and in the normal structure of education in public networks; and the evaluation conducted by blind review concerning the exposure of the student to the intervention are strong aspects of this study.

However, this study also has some limitations. During the analysis, there were four losses in the intervention group and seven losses in the control group. These losses (school transfers), however, do not seem to be related to the fact that the children had these lessons. Before randomization, the parents of 33 children in a total of 118 did not want their children to take part in the project, presenting as justification that they were religious and did not approve of dancing. If this factor had any selection bias, its effect affected both the intervention and the control group. Maybe the intervention should have been pointed out as “games”, “creative activities”, once the techniques of the educational dance methodology are not similar to the popular imaginary of dancing.

Another possible objection would be a contamination bias between the intervention/control groups; however, the nature of the intervention implied a schedule built by a professional specialized in the Laban methodology, with activities elaborated considering progressive levels of difficulty, synchronized with the specific moment of development of the group. The intervention was not reduced to a practice that could be disseminated among the children. This fact makes the risk of contamination between the intervention and the control groups be close to zero.

Finally, the motor development of the children who participated in educational dance lessons was, in average, nine months higher than that of children who did not attend the lessons. However, after six to eight months, this development was equal to that of children who did not participate in the intervention, with the natural evolution of development expected chronologically. A more continuous exposure could maintain the intervention gains.
